# Vasospasm is a significant factor in cyclosporine-induced neurotoxicity: Case report

**DOI:** 10.1186/1471-2377-10-30

**Published:** 2010-05-11

**Authors:** Hilde MH Braakman, Jan Lodder, Alida A Postma, Lambert FR Span, Werner H Mess

**Affiliations:** 1 Department of Neurology, Maastricht University Medical Centre, Maastricht, the Netherlands; 2 Department of Radiology, Maastricht University Medical Centre, Maastricht, the Netherlands; 3 Department of Internal Medicine, Division of Haematology, University Medical Centre Groningen, Groningen, the Netherlands; 4 Department of Clinical Neurophysiology, Maastricht University Medical Centre, Maastricht, the Netherlands

## Abstract

**Background:**

The aetiology of central nervous system lesions observed in cerebral cyclosporine neurotoxicity remains controversial.

**Case presentation:**

We report a 48-year-old woman with a non-severe aplastic anaemia who presented with stroke-like episodes while on cyclosporine treatment.

Transcranial Doppler ultrasound revealed severely elevated flow velocities in several cerebral vessels, consistent with vasospasm. Immediately after reducing the cyclosporine dose, the stroke-like episodes disappeared. Only after cyclosporine withdrawal the transcranial Doppler ultrasound abnormalities fully resolved.

**Conclusions:**

This case demonstrates a significant role of vasospasm in the pathway of cyclosporine-induced neurotoxicity. Transcranial Doppler ultrasound is an effective tool for the diagnosis and follow-up of cyclosporine-induced vasospasm.

## Background

Cyclosporine A is a potent immunosuppressive agent used in various immunological disorders and is administered to prevent graft rejection of transplantations[1]. Neurotoxicity is the second most common side effect of cyclosporine after nephrotoxicity and appears in up to 60% of cyclosporine-treated patients[2]. Since predominantly the central nervous system is affected, the clinical symptoms of cyclosporine-mediated neurotoxicity are decreased responsiveness, hallucinations, delusions, seizures, cortical blindness, aphasia, ataxia and stroke-like episodes. Minor symptoms include tremor, agitation, insomnia, anxiety, amnesia, headache, and paraesthesias[1,2]. Central nervous system lesions caused by cyclosporine are located in the border zones between major vascular territories or their main branches[3]. Lesions can occur even when cyclosporine blood-through levels are within the therapeutic range[2]. The aetiology of the lesions observed in cerebral cyclosporine neurotoxicity remains controversial.

## Case presentation

**Figure 1:**
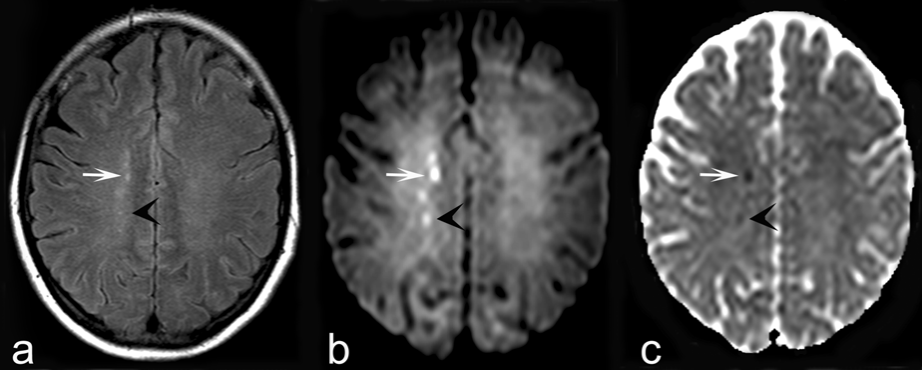
**The axial FLAIR image (1A) shows small hyperintense foci in the watershed area in the right hemisphere**. Some of these foci show high signal intensity on DWI (1B) and low signal intensity at the ADC-map (1C), consistent with restrictive diffusion (arrows).

**Figure 2:**
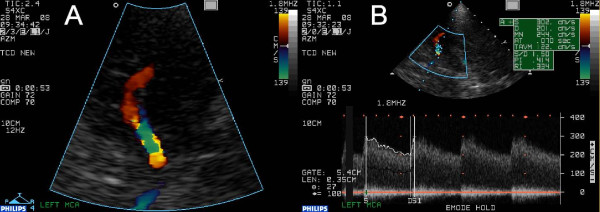
**Initial ultrasound findings**. On figure 1A, the mainstem and one M2 segment of the left middle cerebral artery are depicted in colour mode using transcranial colour coded duplex sonography. Note the typical aliasing in the most proximal segment despite the rather insensitive setting of the colour scale, indicating a severely stenosed segment. On figure 1B, the Doppler spectogram as measured in that segment is given, indeed showing highly elevated blood flow velocities.

**Figure 3:**
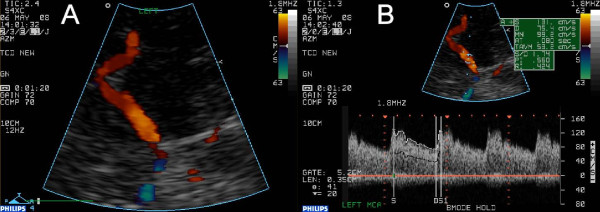
**Ultrasound findings six weeks later**. The aliasing in the most proximal segment is not present anymore despite a more sensitive setting of the colour scale. Accordingly, the Doppler spectogram shows completely normal blood flow velocities.

A 48-year-old woman, with a medical history of a non-severe aplastic anaemia, was re-admitted to the haematology department. She recently had been treated with horse antithymocyte globulin (15 mg/kg/day intravenously for 5 days) in combination with high dose cyclosporine and prednisolone, in order to prevent acute serum sickness. Her current oral medication consisted of cyclosporine 350 mg bidaily (6 mg/kg bidaily), prednisone 60 mg once daily, ciprofloxacin 500 mg bidaily and fluconazole 200 mg once daily. At admission, she presented with stroke-like episodes; repetitive episodes of sudden loss of strength in the left leg and numbness on the left side of the face. The motor symptoms resolved within a few minutes to one hour, although a cold feeling in her left leg and left side of her face persisted. She reported no other symptoms. Her vital signs, including blood pressure (110/70 mmHg), remained within normal range during these episodes. Neurological examination directly after an episode, revealed no abnormalities besides the disturbed temperature sensations. Furthermore, she was bi-cytopenic (haemoglobin level of 6.1 mmol/l with no schistocytes and thrombocyte count of 48 × 10^9^/l) with normal leukocytes (white blood cell count 4.4 × 10^9^/l with 50% granulocytes). Besides the low thrombocyte count, there were no coagulation disorders. Serum calcium level was 2.07 mmol/L (normal range 2.10 mmol/L-2.55 mmol/L) and serum magnesium level was 0.56 mmol/L (normal range 0.70 mmol/L-1.00 mmol/L), for which replenishment was started. Serum cyclosporine concentration was too high (0.64 mg/l, normal range of 0.10-0.30 mg/l); all other laboratory findings, including renal function and cholesterol level, were normal. Electrocardiogram, 24-hour Holter registration and cardiac ultrasonography revealed no abnormalities. Brain magnetic resonance imaging revealed small hyperintense punctuate foci in the watershed region of the right semioval centre up to the subcortical area between the anterior and middle cerebral arteries (Figure 1A). Some of these small foci showed restricted diffusion using diffusion weighted imaging sequences (Figure 1B and 1C). Lumbar puncture was unremarkable. Extracranial duplex examination showed no abnormalities in the carotid and vertebral arteries. Transcranial Doppler ultrasound revealed segments of severely elevated flow velocities in several cerebral vessels, particularly both middle cerebral arteries (Figure 2A and 2B). These findings were consistent with vasospasm. Based on the toxic cyclosporine concentration in absence of schistocytes (fragmented red blood cells) with no signs of acute serum sickness and thrombotic thrombocytopenic purpura, cyclosporine was reduced with 20% and fluconazole and prednisone were withdrawn. Immediately after reducing the cyclosporine dose, the stroke-like episodes disappeared. One week after the dose reduction serum cyclosporine concentration had normalized to 0.29 mg/l, but blood flow velocities as measured with transcranial Doppler ultrasound were practically unchanged. Thereupon, cyclosporine was withdrawn. Six weeks after discontinuation of treatment the transcranial Doppler ultrasound abnormalities had fully resolved (Figure 3A and 3B, and Additional file 1: supplemental table 1), the patient remained asymptomatic, and had remission of aplastic anaemia.

## Discussion

The specific distribution of the increased blood flow velocities measured by transcranial Doppler ultrasound, toxic cyclosporine levels and follow-up in our patient clearly demonstrate a significant role of vasospasm in the pathway of cyclosporine-induced neurotoxicity.

Although clinically unlikely, vasculitis or recanalized stenoses of embolic origin may also cause the transcranial Doppler ultrasound changes.

Previous authors have suggested hypertension, direct neurotoxicity, thrombotic microangiopathy, metabolic disturbances, or a combination of these factors as underlying pathophysiology of cyclosporine-induced neurotoxicity[4]. A distinct clinical entity associated with cyclosporine treatment is the "Posterior reversible encephalopathy syndrome" (PRES). PRES is a syndrome clinically characterized by headache, vomiting, confusion, seizures, cortical blindness and other visual abnormalities, and sometimes motor signs, with focal, often symmetric vasogenic edema as its key radiological and pathologic feature [5-8]. The vasogenic edema is thought to result from epithelial dysfunction with vasoconstriction, leukocyte trafficking, or both[9].

In this patient, we believe cyclosporine-induced vasospasm induced this clinical disease. Magnetic resonance imaging revealed focal restricted diffusion, indicative of ischemia, rather than the vasogenic edema of PRES (Figure 1). If cyclosporine therapy is prolonged, the persisting vasospasms may contribute to the development of true PRES, through prolonged hypoxemia leading to endothelial/cellular vascular endothelial growth factor expression, increased vascular permeability and ultimately vasogenic edema. The role of the latter mechanism was highlighted in a recent case report[10].

Although here used in a setting of aplastic anaemia, these findings most likely pertain to all instances where cyclosporine is used, including stem cell and solid organ transplantation. Patients with aplastic anaemia or allogeneic stem cell transplantations receive higher dosages of cyclosporine than solid organ transplanted patients. Therefore, the former category may be at increased risk of cyclosporine neurotoxicity including PRES vasogenic edema.

Cyclosporine-induced neurotoxicity can present in various distinct clinical syndromes; vasospasm as a primary or secondary phenomenon probably plays a key role. Truwit *et al *were the first who suggested that vasospasm of the intracranial arteries contributed to the mechanism of neurotoxicity. They postulated that endothelial damage plays a primary role, with the release of vasoactive peptides leading to vasospasm as a secondary phenomenon[11]. Later, magnetic resonance angiography was used to assess vasospasm as a central mechanism of cyclosporine neurotoxicity. Interestingly, the lumen narrowing was reversible within weeks after therapy cessation[4,12,13]. A comparable temporal course was observed in our patient and was also seen by Shbarou *et al *who described the first two cases in which, next to magnetic resonance angiography, transcranial Doppler ultrasound was used to assess cyclosporine-induced vasospasm[13].

## Conclusions

Central nervous system dysfunction in patients receiving cyclosporine should alert the neurologist to rule out vasospasm of the main arteries of the circle of Willis. Transcranial Doppler ultrasound is a readily available, effective, non-invasive and low-cost tool for the diagnosis and follow-up of cyclosporine-induced vasospasm. Early diagnosis of cyclosporine neurotoxicity is important. The process of vasospasm is reversible in its early stages but in later stages it may become irreversible or even fatal[2].

## Competing interests

The authors declare that they have no competing interests.

## Authors' contributions

HMHB collected the data and designed and drafted the manuscript. JL helped to draft the manuscript. AAP analysed the MRI data, drafted the MRI figure legends and helped to draft the manuscript. LFRS participated in the data collection, the design of this article and helped to draft the manuscript. WHM participated in the data collection, the design of this article, helped to draft the manuscript and drafted the transcranial Doppler ultrasound figure legends. All authors read and approved the final manuscript.

## Consent

Informed consent was obtained from the patient for publication of this case report and any accompanying images.

## Pre-publication history

The pre-publication history for this paper can be accessed here:

http://www.biomedcentral.com/1471-2377/10/30/prepub

## Supplementary Material

Additional file 1 **Blood flow velocities measured with transcranial Doppler ultrasound**. Blood flow velocities measured with transcranial Doppler ultrasound during cyclosporine treatment (A), One week after cyclosporine dose reduction (B), and six weeks after discontinuation of cyclosporine treatment (C). Click here for file
