# Supplementary material for: Vasospasm is a significant factor in cyclosporine-induced neurotoxicity: Case report
Source: BMC Neurol. 2010 May 11;10:30. doi: 10.1186/1471-2377-10-30 (PMC2874792; doi:10.1186/1471-2377-10-30)
Supplement: Additional file 1 — Blood flow velocities measured with transcranial Doppler ultrasound. Blood flow velocities measured with transcranial Doppler ultrasound during cyclosporine treatment (A), One week after cyclosporine dose reduction (B), and six weeks after discontinuation of cyclosporine treatment (C). [file 1471-2377-10-30-S1.DOC]

Table 1. Blood flow velocities measured with transcranial Doppler ultrasound during cyclosporine treatment (A), One week after cyclosporine dose reduction (B), and six weeks after discontinuation of cyclosporine treatment (C)

A

| Right | depth [mm] | syst [cm/s] | diast [cm/s] | mean [cm/s] | p.i. | remarks |
| --- | --- | --- | --- | --- | --- | --- |
| middle cerebral artery  anterior cerebral artery  posterior cerebral artery    posterior communicating artery | 37  42  52  50  57  60  63  66  60 | 100  171  258  309  251  114  122  78  116 | 55  115  170  218  168  77  79  47  78 | 80  146  206  262  201  97  95  60  99 | 0.6  0.4  0.4  0.3  0.4  0.4  0.5  0.5  0.4 | turbulent signal  musical murmurs  P1  P2  open |

| Left | depth [mm] | syst [cm/s] | diast [cm/s] | mean [cm/s] | p.i. | remarks |
| --- | --- | --- | --- | --- | --- | --- |
| middle cerebral artery  anterior cerebral artery  posterior cerebral artery  posterior communicating artery | 39  44  51  53  54  56  59  65  66  59 | 175  143  320  296  302  357  248  141  93  146 | 124  97  224  199  201  250  168  85  68  91 | 152  274  239  244  301  202  111  81  115 | 0.3  0.4  0.4  0.4  0.4  0.4  0.5  0.3  0.5 | turbulent signal  P1  P2  open |

B

| Right | depth [mm] | syst [cm/s] | diast [cm/s] | mean [cm/s] | p.i. | remarks |
| --- | --- | --- | --- | --- | --- | --- |
| middle cerebral artery  anterior cerebral artery  posterior cerebral artery | 52  55  59  61  67  68  59 | 262  287  205  189  91  107  146 | 157  168  127  127  52  70  93 | 193  204  161  152  67  87  116 | 0.5  0.6  0.5  0.4  0.6  0.4  0.5 | turbulent signal in all parts of the vessel  P1  P2 |

| Left | depth [mm] | syst [cm/s] | diast [cm/s] | mean [cm/s] | p.i. | remarks |
| --- | --- | --- | --- | --- | --- | --- |
| middle cerebral artery  anterior cerebral artery  posterior cerebral artery | 39  47  50  55  59  65  67  60 | 128  135  309  290  228  130  72  60 | 79  77  199  187  139  77  47  34 | 101  103  247  230  180  96  56  45 | 0.5  0.6  0.4  0.4  0.5  0.6  0.4  0.6 | turbulent signal in all parts of the vessel  musical murmurs  P1  P2 |

C

| Right | depth [mm] | syst [cm/s] | diast [cm/s] | mean [cm/s] | p.i. | remarks |
| --- | --- | --- | --- | --- | --- | --- |
| middle cerebral artery  anterior cerebral artery  posterior cerebral artery  posterior communicating artery | 37  44  52  56  62  68  56  63  65 | 78  76  108  114  85  52  67  90  83 | 48  39  67  58  47  31  39  52  43 | 63  55  85  85  64  41  54  69  60 | 0.5  0.7  0.5  0.7  0.6  0.5  0.5  0.6  0.7 | open |

| Left | depth [mm] | syst [cm/s] | diast [cm/s] | mean [cm/s] | p.i. | remarks |
| --- | --- | --- | --- | --- | --- | --- |
| middle cerebral artery  anterior cerebral artery  posterior cerebral artery  posterior communicating artery | 39  48  52  53  56  71  65 | 63  90  131  123  125  96  61 | 31  46  75  64  67  52  34 | 44  67  99  90  91  69  49 | 0.7  0.7  0.6  0.7  0.6  0.6  0.6 | open |

p.i.= pulsatility index
